# Multiple E3s promote the degradation of histone H3 variant Cse4

**DOI:** 10.1038/s41598-017-08923-w

**Published:** 2017-08-17

**Authors:** Haili Cheng, Xin Bao, Xin Gan, Shiwen Luo, Hai Rao

**Affiliations:** 1 Department of Molecular Medicine, The University of Texas Health Science Center, San Antonio, TX 78229 USA; 2 Research Institute of Respiratory Medicine, The First Affiliated Hospital, Nanchang University, Nanchang, China; 3 Center for Experimental Medicine, The First Affiliated Hospital, Nanchang University, Nanchang, China

## Abstract

The histone H3-like protein Cse4/CENP-A acts as a key molecular marker that differentiates the special centromeric chromatin structures from bulk nucleosomes. As altered Cse4/CENP-A activity leads to genome instability, it is pivotal to understand the mechanism underlying Cse4 regulation. Here, we demonstrate that four ubiquitin ligases (i.e., Ubr1, Slx5, Psh1, and Rcy1) work in parallel to promote Cse4 turnover in yeast. Interestingly, Cse4 overexpression leads to cellular toxicity and cell cycle delay in yeast cells lacking *PSH1*, but not in cells lacking *UBR1*, suggesting different roles of these two degradation pathways. Our findings suggest that various ubiquitin ligases collaborate to keep the Cse4 level in check, providing a basis for further delineating the intricate network involved in Cse4 regulation.

## Introduction

The centromere, a highly condensed and constricted chromosome region, serves as the site of kinetochore assembly, which is essential to chromosome segregation that allows proper distribution of duplicated genetic information to daughter cells^1, 2^. The yeast histone H3 variant Cse4 and its human counterpart CENP-A are molecular markers that differentiate the special centromeric structure from bulk nucleosomes^2–5^. Excessive or nonrestraint Cse4/CENP-A can lead to its incorporation into euchromatic nucleosomes, which may result in aneuploidy and promote tumorigenesis^2, 6–8^. It is therefore critical to understand how Cse4/CENP-A activity is regulated and restricted to the centromere.

Human CENP-A and yeast Cse4 have been shown to be subject to ubiquitin-mediated degradation^2, 9, 10^. Ubiquitin, a 76-residue polypeptide, often functions as the molecular flag that marks protein for destruction, promoting rapid changes in protein concentration^11–13^. The key factors in ubiquitin-mediated events are the ubiquitin ligases (E3s), which specifically recognize substrates. With the help of a ubiquitin-activating enzyme (E1) and a ubiquitin-conjugating enzyme (E2), E3 ligases facilitate the covalent attachment of ubiquitin molecules onto their targets. Ubiquitin-decorated substrates are then transferred to and degraded by the 26S proteasome, a multi-subunit protease^11–13^. As E3 ligases confer substrate selectivity and carry out the rate-limiting step of ubiquitylation, the key to understanding the role of ubiquitin-mediated proteolysis in Cse4/CENP-A regulation is identifying ubiquitin ligases that are responsible for selecting Cse4/CENP-A for ubiquitylation and degradation.

The first degradation route for Cse4 was identified ~7 years ago but only accounted for partial Cse4 degradation. Using an affinity purification-mass spectrometry coupled strategy, Psh1 was shown to bind Cse4 directly and promote its ubiquitylation^14, 15^. We recently performed a synthetic dosage lethality screen and identified the SCF (Skp1-Cdc53-F box) complex containing the F-box protein Rcy1 as another ubiquitin ligase required for Cse4 turnover^16^. Excessive Cse4 accumulation leads to growth retardation in cells lacking *PSH1* or *RCY1*. However, in the absence of both *PSH1* and *RCY1*, Cse4 is still degraded albeit more slowly^16^, suggesting other degradation pathway(s) remains to be discovered.

To uncover other ubiquitylation enzymes involved in Cse4 regulation, we screened for ubiquitin-conjugating enzymes (E2) involved and found that Cse4 turnover is impaired in yeast cells lacking *UBC4*. We next demonstrated that a Ubc4-dependent E3 complex composed of Slx5 and Slx8 promotes Cse4 turnover. Moreover, we showed that Ubr1 is the fourth E3 involved in Cse4 ubiquitylation and degradation. Our results suggest that these four ubiquitin ligases likely act in parallel to maintain the Cse4 level. Interestingly, unlike *PSH1*-defective cells, *UBR1* deficiency does not lead to growth retardation and cell cycle delay upon Cse4 overexpression. These findings offer novel insights into the mechanism underlying Cse4/CENP-A regulation.

## Results

### The Ubc4-Slx5-Slx8 pathway is involved in Cse4 turnover

**Figure 1:**
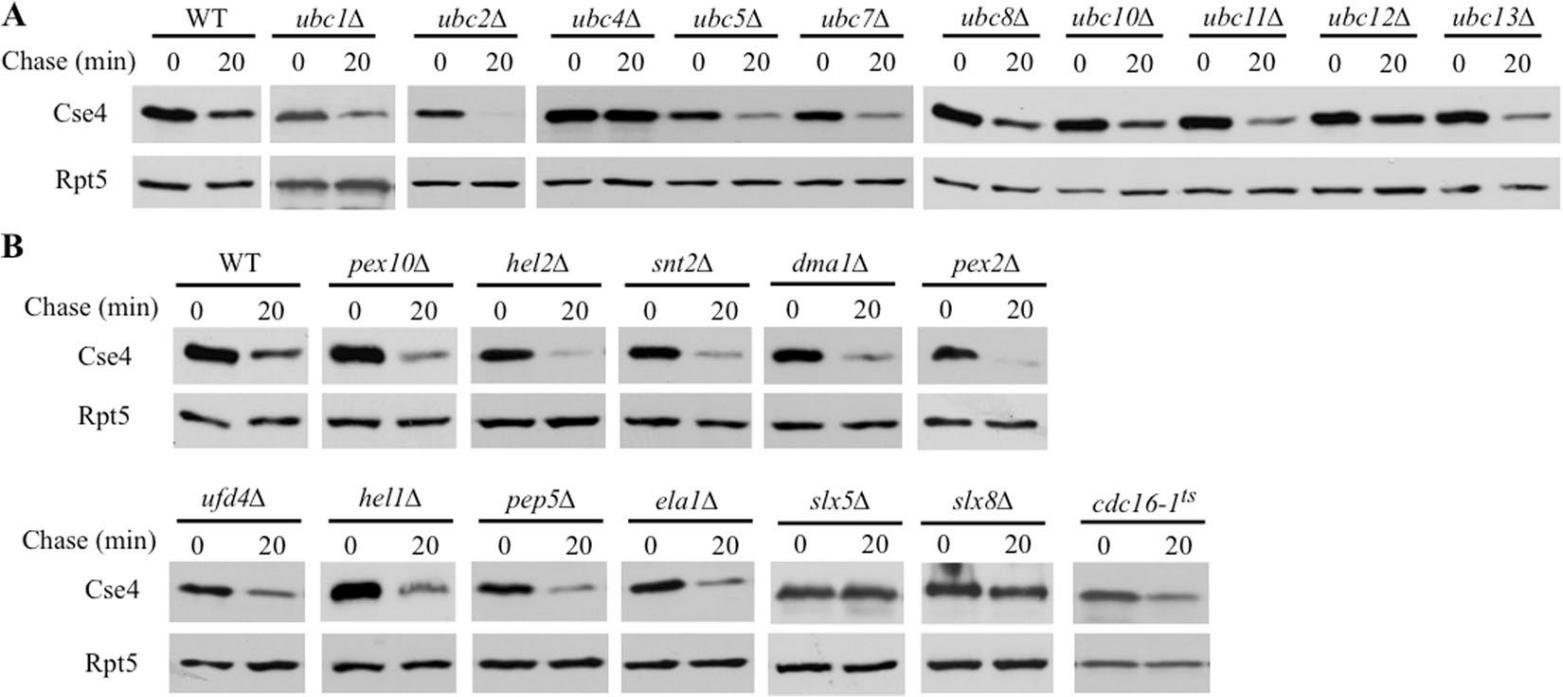
The involvement of Ubc4 E2 and Ubc4-associated E3s in Cse4 degradation. (**A**) Cse4 turnover is impaired in *ubc4Δ* cells. Cse4 stability was examined in wild-type and E2-deficient cells by a protein expression shut-off assay. A plasmid bearing HA-tagged Cse4 was transformed into wild-type cells and 10 E2 mutants. Yeast cells expressing HA-Cse4 were grown to an OD_600_ of ~1, and samples were collected after expression was turned off by cycloheximide (indicated as Chase (min). Extracts were analyzed by immunoblotting with HA antibody. Equal amounts of extracts were used and verified by western blotting with Rpt5 antibody in all of protein stability experiments (lower panels). (**B**) Efficient Cse4 degradation requires Slx5 and Slx8. Cse4 turnover in wild-type cells and 12 Ubc4-associated E3 mutant cells was determined as above.

**Figure 2:**
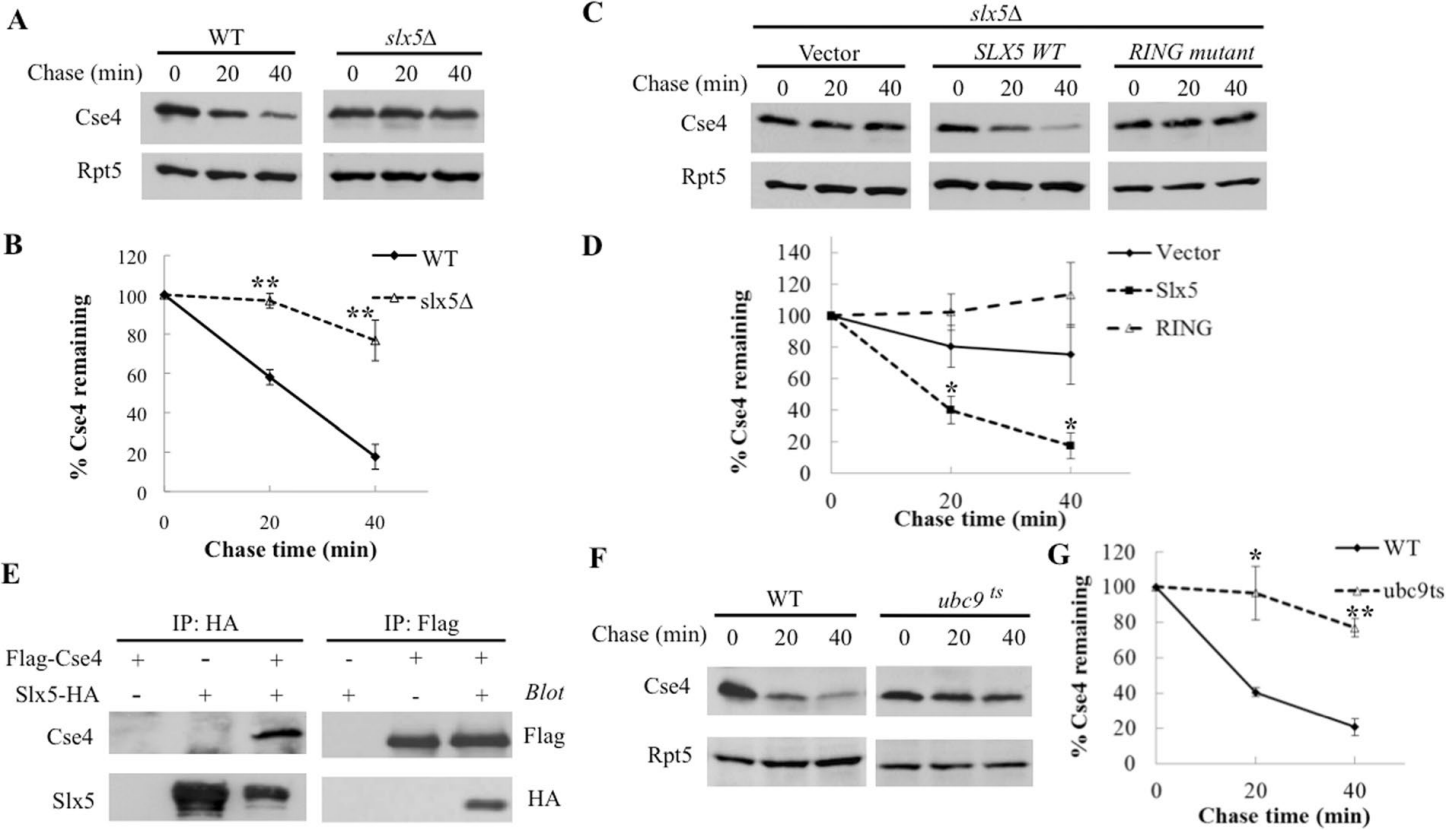
The Slx5 pathway promotes Cse4 turnover. (**A**) Cse4 degradation is impaired in yeast cells lacking *SLX5*. Cse4 stability in *slx5Δ* cells was determined as described above. (**B**) Quantitation of the data in **A**. The experiments were performed more than 3 times, and the average values with S.D. are presented. P values determined by a two tailed Student’s t-test are indicated, *p < 0.05, **p < 0.005. (**C**) An intact RING finger domain of Slx5 is required for Cse4 degradation. *slx5Δ* cells bearing HA-Cse4 were transformed with a control vector, or a plasmid expressing wild-type Slx5 or a Slx5 RING mutant as indicated. Cse4 turnover was then monitored. (**D**) Quantitation of the data in **C**. (**E**) Slx5 interacts with Cse4. Plasmids bearing HA-tagged Slx5 and/or Flag-Cse4 were transformed into wild-type yeast cells. Proteins were extracted and incubated with the IgG beads coated with HA or Flag antibody. The samples were resolved by SDS-PAGE and visualized by western blotting using Flag or HA antibody as indicated. The identity of the bands is shown to the left of the panels. The antibodies used for IP and western blotting are indicated. (**F**) Ubc9 is involved in Cse4 destruction. (**G**) Quantitation of the data in **F**.

Ubiquitin-mediated pathways are mainly defined by specific ubiquitin-conjugating enzymes (E2) and ubiquitin ligases (E3)^13^. In the yeast *S. cerevisiae*, there are ~12 E2s and more than 60 E3s involved in ubiquitin modification^11^. To uncover other pathways responsible for Cse4 turnover, we first sought to identify relevant E2s. Ubc3 (also called Cdc34) has been previously implicated as an E2 for Cse4^17^. Here, we assessed Cse4 degradation kinetics in yeast cells lacking one of the ten non-essential E2 enzymes (i.e., Ubc1, Ubc2, Ubc4, Ubc5, Ubc7, Ubc8, Ubc10, Ubc11, Ubc12, and Ubc13). Interestingly, Cse4 turnover was impaired in the *ubc4Δ* mutant (Fig. 1A), suggesting that Ubc4 functions as another E2 enzyme in Cse4 regulation. This finding prompted us to examine whether known Ubc4-associated E3s (e.g., Ufd4, Dma1, Hel1, Ela1, Slx5, and APC) are involved in Cse4 turnover (Fig. 1B). We found that an E3 complex composed of Slx5 and Slx8 was required for efficient Cse4 degradation (Figs 1B and 2A,B). The compromised Cse4 turnover in the *slx5Δ* mutant could be restored by the expression of wild-type Slx5, but not the RING mutant of Slx5 (C561S C564S) deficient in ubiquitylation, suggesting a requirement for Slx5 E3 catalytic activity in Cse4 degradation (Fig. 2C,D).

As an E3 directly interacts with its target, we evaluated whether Slx5 binds Cse4 *in vivo* by co-immunoprecipitation assays (Fig. 2E). We found that Slx5 and Cse4 reciprocally co-immunoprecipitated (Fig. 2E), supporting the involvement of Slx5 in Cse4 regulation. Slx5 is known as a SUMO-targeted ubiquitin ligase (STUbL) that recognizes sumoylated proteins as substrates. Slx5 substrates are first sumoylated by the Ubc9 (E2)-Siz1/2 (E3) pathway, then recognized and ubiquitylated by the Ubc4 (E2)-Slx5-Slx8 (E3) pathway for subsequent degradation. We therefore wondered whether sumoylation was involved in Cse4 degradation. Ubc9 is the sole and essential SUMO conjugating enzyme in yeast. When a yeast strain bearing a temperature sensitive mutation in *UBC9* was shifted from 30 °C to the non-permissive temperature 37 °C, Cse4 degradation was markedly compromised (Fig. 2F,G), which is consistent with the link between sumoylation and Slx5 in proteolysis.

While our work was in progress, Ohkuni *et al*. also demonstrated the involvement of Slx5 and Slx8 in Cse4 degradation^18^. Using a different approach (i.e., post-translational modification of Cse4), they established Cse4 sumoylation *in vivo* and *in vitro* via the SUMO ligases Siz1 and Siz2, which then attracts the Slx5-Slx8 E3 complex for subsequent substrate ubiquitylation and degradation^18^. Our results complement their findings and further reveal an *in vivo* involvement of the E2 enzymes Ubc4 and Ubc9 in Cse4 regulation (Figs 1 and 2).

### Cse4 degradation is compromised in cells lacking Psh1, Slx5 and Rcy1

**Figure 3:**
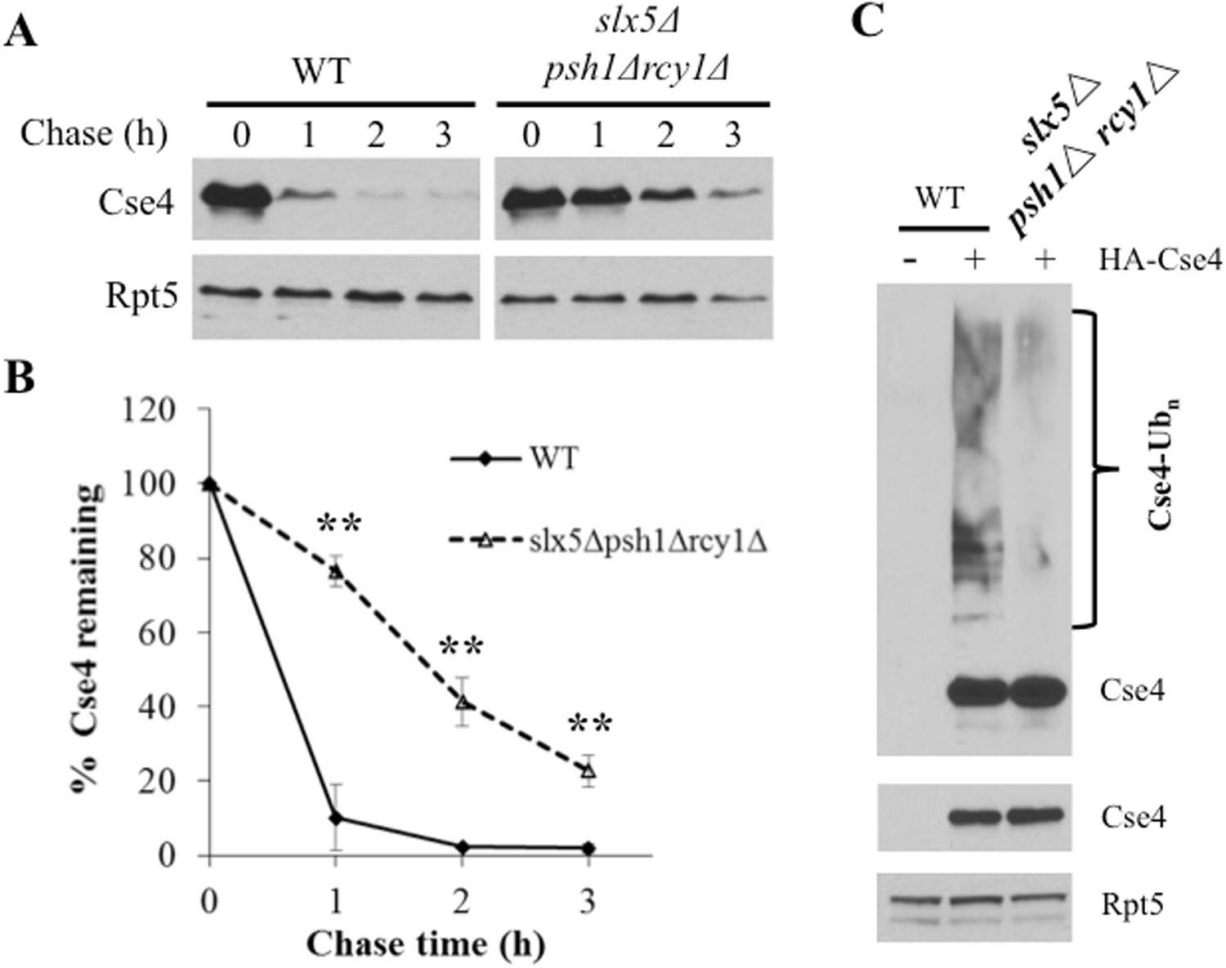
Cse4 ubiquitylation and degradation are impaired in the triple mutant *psh1Δ rcy1Δ slx5Δ* cells. (**A**,**B**) Cse4 stability in wild-type and *psh1Δ rcy1Δ slx5Δ* cells was determined and quantified as in Fig. 2. (**C**) Cse4 ubiquitylation is reduced in *psh1Δ rcy1Δ slx5Δ* mutant cells. Briefly, wild-type or mutant yeast cells bearing a vector control plasmid or a plasmid expressing HA-Cse4 were grown in galactose media for 4 hours for Cse4 induction. Ubiquitylated Cse4 species were enriched by incubating extracts with tandem ubiquitin binding entities (TUBEs) agarose gel. Cse4 and its ubiquitin conjugates were detected by immunoblotting with anti-HA. Ubiquitylated and unmodified Cse4 are indicated to the right of the panel. To ensure equal loading, the amounts of Cse4 and the control protein Rpt5 in the extracts were determined and are shown in the lower panels.

As both double mutants *psh1Δ slx5Δ* and *psh1Δ rcy1Δ* exhibit a slower Cse4 degradation rate than single mutants (i.e., *psh1Δ*, *rcy1Δ*, *slx5Δ*)^16, 18^, Psh1 is thought to operate independently of Slx5 and Rcy1.We also found that Slx5 and Rcy1 function in separate pathways (see below in Fig. 6B). Next, we examined whether Cse4 degradation would be abolished in yeast cells missing all three of these ubiquitin ligases Psh1, Slx5 and Rcy1. Instead of using shorter time points up to 40 minutes, which help to capture moderate effects, we extended the chase time of Cse4 stability experiment to 3 hours (Fig. 3A). Interestingly, Cse4 turnover was severely impaired but not entirely eliminated in the *psh1Δ rcy1Δ slx5Δ* triple mutant (Fig. 3A,B), suggesting that there are other degradation pathways for Cse4. Consistent with the observation of compromised Cse4 turnover in the *psh1Δ rcy1Δ slx5Δ* mutant, Cse4 ubiquitylation was also significantly reduced in the triple mutant as well (Fig. 3C).

### Ubr1 E3 promotes Cse4 ubiquitylation and degradation

**Figure 4:**
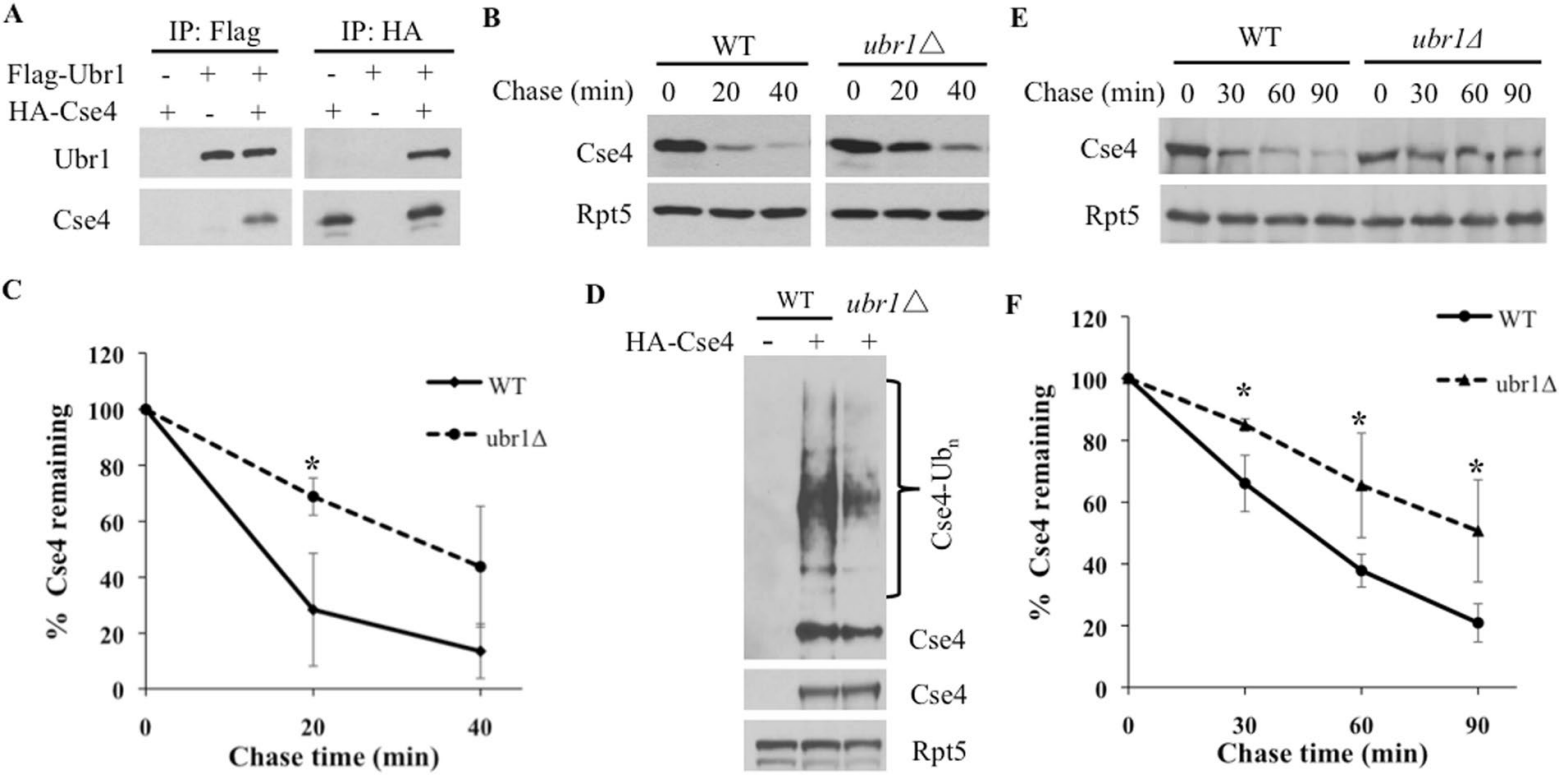
Ubr1 E3 regulates Cse4 turnover. (**A**) Ubr1 associates with Cse4 *in vivo*. A plasmid bearing HA-Cse4 or Flag-Ubr1 and/or a control vector were transformed into yeast cells. Proteins were extracted from the cells indicated and incubated with IgG beads coated with Flag or HA antibody. The samples were resolved by SDS-PAGE and visualized by western blotting using Flag or HA antibody as indicated. The identity of the bands is shown to the left of the panels. (**B**,**C**) Cse4 degradation in wild-type and *ubr1Δ* cells was determined and quantified as above. (**D**) Reduced Cse4 ubiquitylation in *ubr1Δ* cells. (**E**) Ubr1 is involved in the degradation of endogenously expressed Cse4-GFP. Expression shut-off analysis of Cse4 tagged with GFP at its normal chromosomal locus was carried out in wild-type and *ubr1Δ* cells. Cse4-GFP was immunoprecipitated by a GFP antibody and then analyzed by western blotting. (**F**) Quantitation of the data in **E**.

To identify other ubiquitin ligases involved, we re-examined the list of candidate Cse4-binding proteins that were previously isolated using an immunoprecipitation-mass spectrometry approach^15^. Besides Psh1^15^, Ubr1 was the only other E3 obtained in the screen but its potential role in Cse4 regulation had not been characterized. Ubr1 was the first E3 ligase identified in ubiquitin-mediated processes and has many substrates localized in the nucleus and cytosol^19, 20^. First, we performed co-immunoprecipitation to validate the Cse4-Ubr1 interaction. We found that Flag-tagged Ubr1 and HA-tagged Cse4 immunoprecipitated each other (Fig. 4A), supporting binding between Ubr1 and Cse4. Moreover, Cse4 degradation and ubiquitylation are impaired in yeast cells lacking *UBR1* (Fig. 4B,C,D), suggesting that Ubr1 acts as another E3 enzyme for Cse4 turnover. One concern is that Cse4 was overexpressed in these experiments. Therefore, we next employed a strain expressing Cse4 tagged with GFP at its endogenous locus. Due to its lower expression level, GFP-Cse4 was enriched by immunoprecipitation with a GFP antibody in these experiments (Fig. 4E). We found that degradation of endogenously expressed GFP-Cse4 was impaired in cells lacking *UBR1* (Fig. 4E,F), supporting that Ubr1 also participates in Cse4 regulation.

### Differential responses of *psh1Δ* and *ubr1Δ* cells to Cse4 overexpression

**Figure 5:**
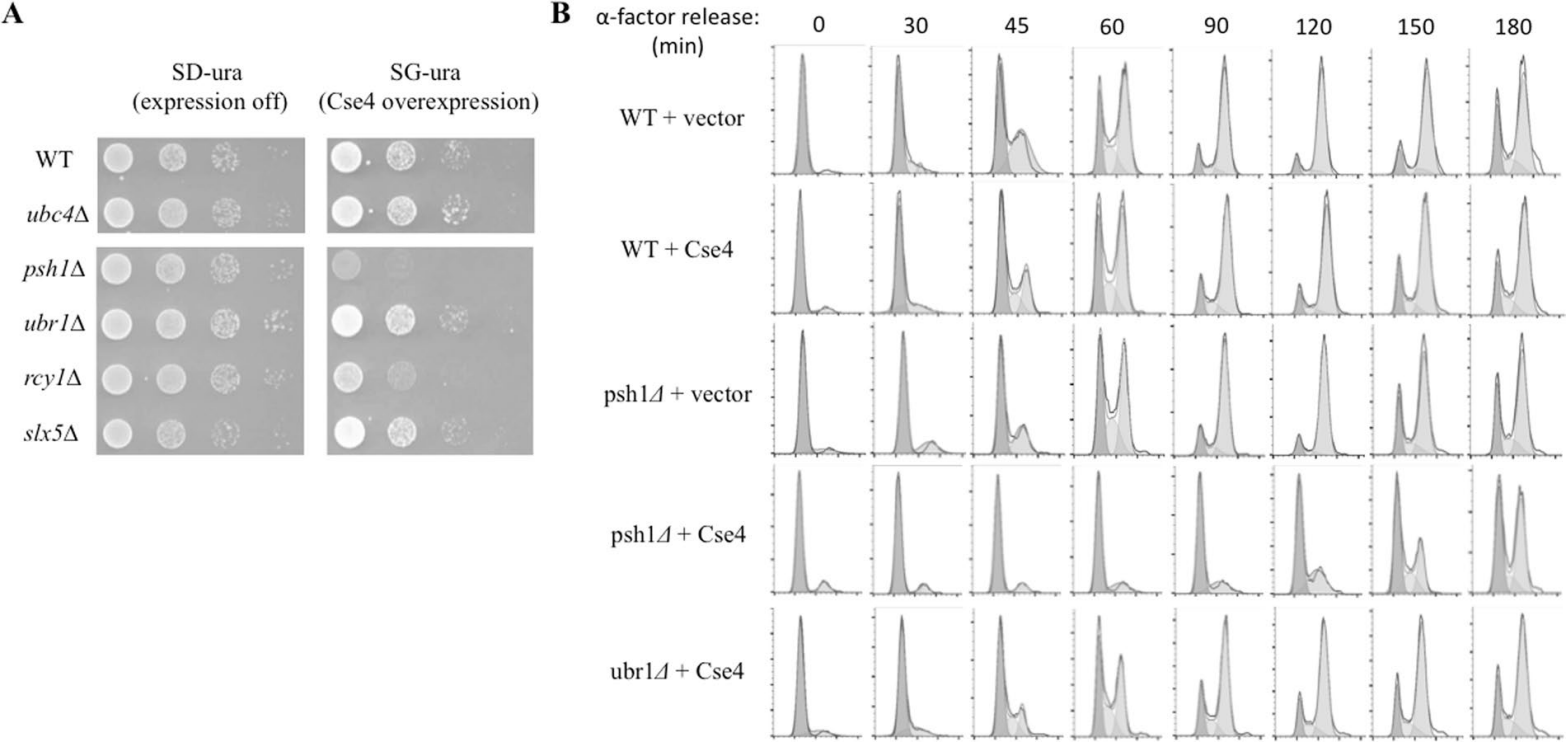
Cse4 overexpression leads to toxicity and cell cycle delay in *psh1Δ* but not *ubr1Δ* mutant cells. (**A**) Cse4 overexpression causes slower growth in cells lacking *PSH1* or *RCY1*. A plasmid expressing *GAL1* promoter-regulated Cse4 was transformed into wild-type, *ubc4Δ*, *psh1Δ*, *ubr1Δ*, *rcy1Δ*, *slx5Δ* cells as indicated. Yeast cells were grown to similar densities and 10-fold serial dilutions were spotted onto both glucose (SD, expression off) and galactose (SG, expression on) plates. The identities of these mutants are listed to the left of the panels. (**B**) Effects of Cse4 overexpression on cell cycle progression monitored by FACS. Cells were arrested in G1 triggered by α factor for 4 h. Cse4 expression was induced for 2 h in G1 arrested cells. The cells were released from G1 arrest and maintained in galactose-containing media. The samples collected were subject to FACScan analysis of DNA content. Representative FACS results are shown.

Cse4 accumulation has been shown to cause severe growth inhibition in yeast cells lacking *PSH1* or *RCY1* ^14–16^. Interestingly, Cse4 overexpression has little effect on the growth of *ubr1Δ* or *slx5Δ* cells, unlike mutant cells missing *PSH1* or *RCY1* (Fig. 5A)^14–16^. Consistent with the normal growth observed in *slx5Δ* upon Cse4 induction, cells lacking *UBC4* were not sensitive to Cse4 overexpression (Fig. 5A) as Ubc4 and Slx5 function in the same pathway^18^. To further understand the toxicity triggered by Cse4 accumulation in *psh1Δ* cells, we monitored cell cycle progression by FACS analysis since Cse4-mediated chromosome segregation is a crucial cell cycle event. Wild-type or mutant yeast cells bearing a plasmid for Cse4 overexpression or a vector control were arrested at G1 phase by α-factor and then released to resume normal cell growth. In the absence of Cse4 overexpression, largely similar cell cycle progression kinetics was observed in wild-type, *psh1Δ* (Fig. 5B), and *ubr1Δ* cells (data not shown). However, compared with wild-type cells, *psh1Δ* cells overexpressing Cse4 exhibited a significantly slower cell cycle, showing ~100 minutes delay (Fig. 5B). In contrast, *ubr1Δ* cells overexpressing Cse4 showed only a minor cell cycle delay compared with wild-type cells (Fig. 5B).

### Four E3s work in parallel to promote Cse4 turnover

**Figure 6:**
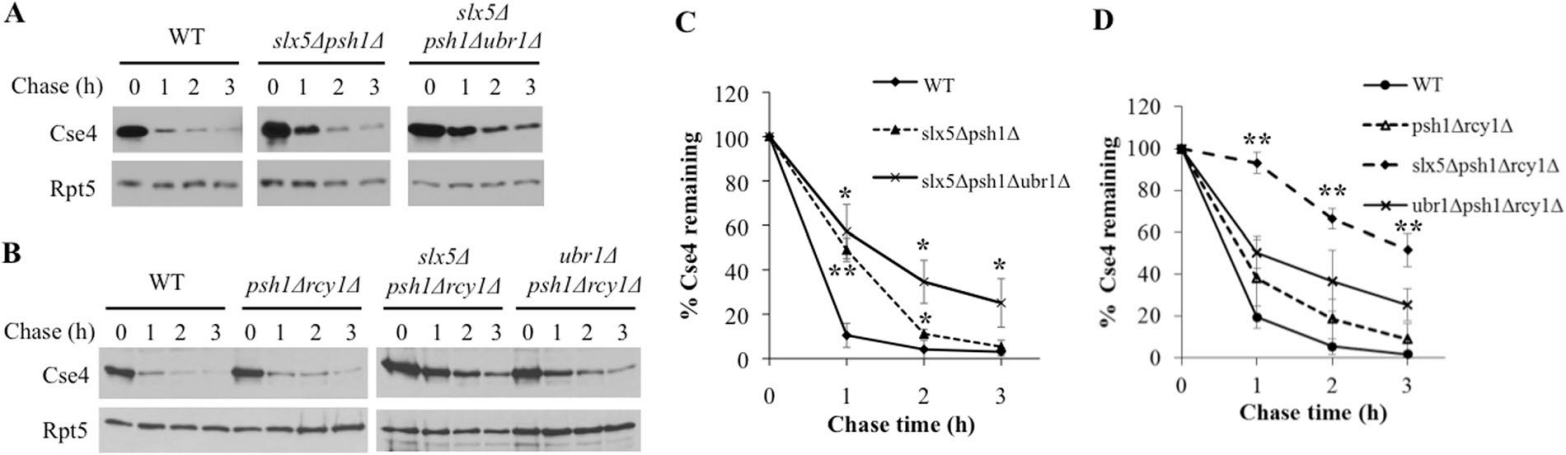
Functional relationships among Ubr1, Slx5, Psh1 and Rcy1. (**A–D**) Cse4 stability was determined and quantified in wild-type and mutant cells bearing various combinations of *rcy1Δ*, *psh1Δ*, *slx5Δ*, *ubr1Δ*.

We then evaluated the relationship between Ubr1 and the other three E3s. Slx5 and Psh1 define two distinct pathways for Cse4 turnover based on a previous study^18^. Therefore, we compared the degradation kinetics of Cse4 in wild-type cells and *psh1Δ slx5Δ*, and *psh1Δ slx5Δ ubr1Δ* mutants using an expression shut-off assay. The triple mutant *psh1Δ slx5Δ ubr1Δ* showed greater impairment of Cse4 turnover than the *psh1Δ slx5Δ* double mutant (Fig. 6A,C), suggesting that Ubr1 likely works in a pathway separate from Psh1 and Slx5.

We previously demonstrated that Rcy1 and Psh1 act in different pathways to promote Cse4 destruction^16^. We found that the triple mutants, *psh1Δ rcy1Δ slx5Δ* and *psh1Δ rcy1Δ ubr1Δ*, showed more compromised Cse4 turnover than the *psh1Δ rcy1Δ* double mutant (Fig. 6B,D), suggesting that Ubr1 and Slx5 work separately, but in parallel with Psh1 and Rcy1. Combined, our results suggest that these E3s define four distinct routes of Cse4 degradation.

**Figure 7:**
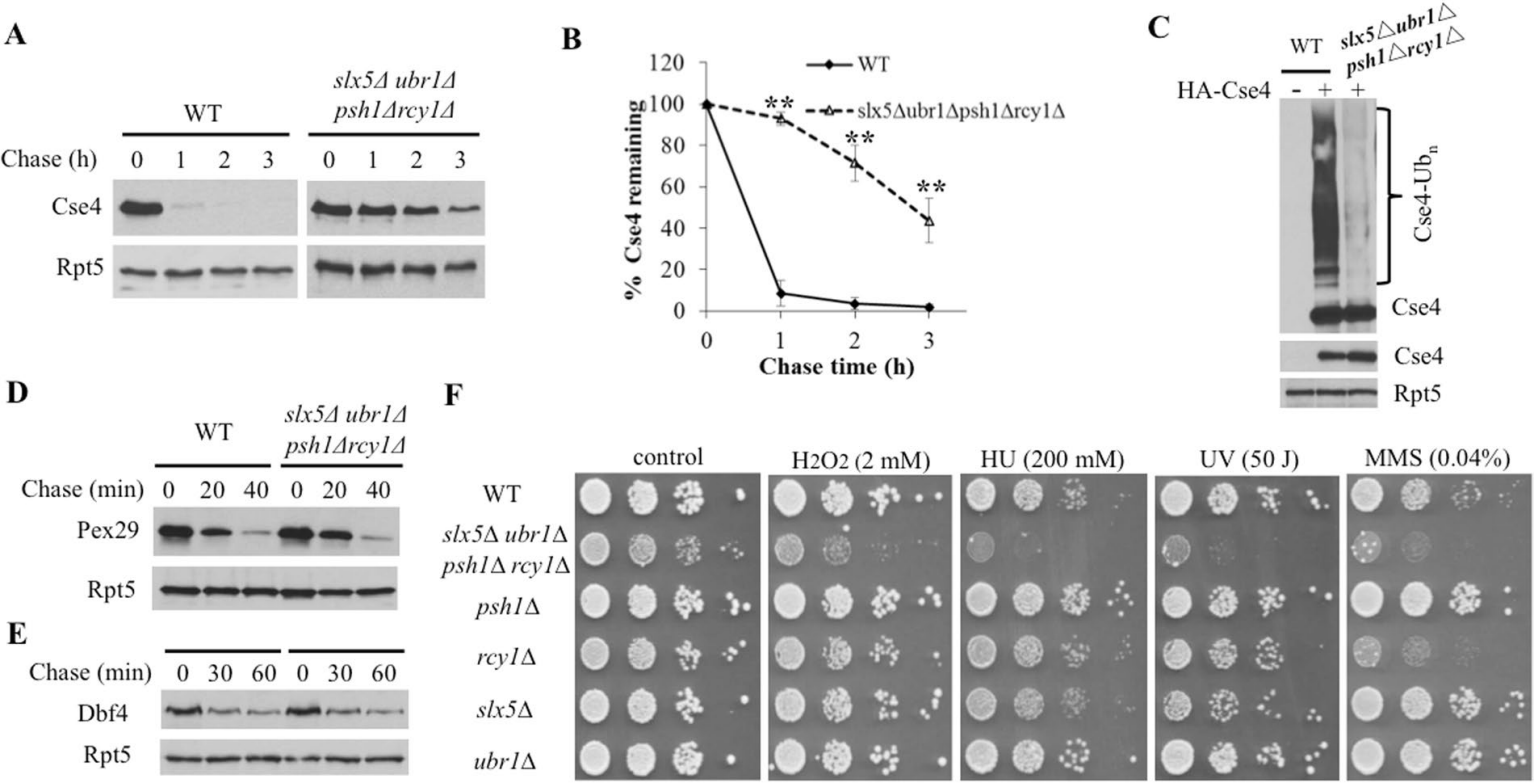
Impaired Cse4 degradation and ubiquitylation in the *psh1Δ rcy1Δ slx5Δ ubr1Δ* quadruple mutant cells. (**A**,**B**) Cse4 degradation in wild-type and *psh1Δ rcy1Δ slx5Δ ubr1Δ* cells was determined and quantified as above. (**C**) Cse4 ubiquitylation was determined as described in Fig. 3C. (**D**,**E**) Degradation of Pex29 and Dbf4 in wild-type and *psh1Δ rcy1Δ slx5Δ ubr1Δ* cells. Pex29 and Dbf4 are regulated by the E3s Doa10 and APC, respectively^31^. The degradation kinetics of GST-tagged Pex29 and MORF-tagged Dbf4 were determined as previously described^31^. (**F**) Phenotypes associated with the quadruple mutant *psh1Δ rcy1Δ slx5Δ ubr1Δ*. Mutants display stress-sensitive phenotypes. Yeast wild-type cells and mutants lacking one or four E3s were spotted in 10-fold serial dilution onto YPD plates with the indicated agents and concentrations.

We further assessed whether these four E3s account for all Cse4-related degradation pathways. Cse4 was significantly stabilized in the quadruple mutant *psh1Δ rcy1Δ slx5Δ ubr1Δ*, but residual degradation remained (Fig. 7A,B), suggesting that additional ubiquitin ligase(s) or a small pool of stable Cse4 may exist. Consistent with this finding, Cse4 ubiquitylation is largely but not entirely eliminated in *psh1Δ rcy1Δ slx5Δ ubr1Δ* mutant cells (Fig. 7C). In contrast to the impaired Cse4 turnover observed in the quadruple mutant *psh1Δ rcy1Δ slx5Δ ubr1Δ*, the degradation of two proteasomal substrates Pex29 and Dbf4 was unaltered in cells lacking these E3s (Fig. 7D,E), indicating that global proteolysis is not affected in the quadruple mutant cells.

We also examined the growth of the quadruple mutant *psh1Δ rcy1Δ slx5Δ ubr1Δ* under various conditions (Fig. 7F). Interestingly, the quadruple mutant is more sensitive than single E3 mutants to DNA-damaging agents (e.g., UV and HU) and oxidative stress triggered by H_2_O_2_ (Fig. 7F), supporting the involvement of these E3s in genome maintenance.

## Discussion

Cse4 plays a crucial role in establishing the centromeric domain, which is key to chromosome segregation^2–5^. Unrestrained Cse4 activity leads to aberrant chromosome-related processes and genome instability^2, 6–8^. Here, we show that at least four E3 ligases (i.e., Psh1, Ubr1, Slx5, Rcy1) work together to regulate the Cse4 level. Although one E3 ligase may be sufficient to adjust the concentration of its targets, multiple ubiquitin ligases have increasingly been found to be involved in modulating a substrate in response to various stimuli. For example, five E3s (i.e., Tom1, Snt2, Pep5, Hel1 and Hel2) have been implicated in the degradation of histone H3^21^. The use of multiple E3s in substrate turnover allows for more regulation and better control. Instead of simply acting like an “on/off” switch, E3s serve more like a dimmer-type control to modulate protein concentration and activity.

How these E3s collaborate remains far from clear. The delayed cell cycle progression observed in *psh1Δ* cells (Fig. 5B) further suggests that sustained Cse4 may activate cell cycle checkpoints to ensure proper chromosome segregation. Our findings raise many challenging issues that would be important for future studies, including how these E3s specifically recognize Cse4, how they work with other cellular cues and pathways (e.g., casein kinase 2, Siz1- and Siz2-mediated sumoylation, SWI/SNF remodeling enzymes, the FACT complex, and the proline isomerase Fpr3)^17, 18, 22–25^, and what the functional role of each degradation pathway is.

The four ubiquitin ligases involved in Cse4 turnover have been identified via different approaches, including immunoprecipitation-mass spectrometry, a synthetic lethality screen, and a systematic analysis for specific E2s and related E3s^14–16, 18^. Since Cse4 degradation is not entirely abolished in the quadruple mutant (Fig. 7), it is possible that a small pool of Cse4 may be spared from degradation or additional ubiquitin ligases are involved in Cse4 turnover. Key to understanding the physiological functions of the ubiquitin system is to delineate the functional relationships between ubiquitin E3 ligases and cognate targets. However, due to the transient nature of substrate-E3 binding and the technical challenges involved, it can be a significant undertaking to identify a relevant E3 involved in the modification of a given substrate. For example, more than 20 years after the discovery of the ubiquitylated form of histone H2B, Bre1 was identified as the ubiquitin ligase in H2B regulation^26, 27^. Identifying cellular targets of an E3 can be equally frustrating as illustrated by the time it took (i.e., ~15 years) to discover a physiological substrate (i.e., Scc1) of the N-end rule E3 Ubr1, the first E3 ligase identified in ubiquitin-mediated events^19, 28^. Given the critical role of Cse4 in centromere function, it may be worthwhile to comprehensively analyze Cse4 stability in all yeast mutants that are defective in E3 activity.

The ubiquitin ligase(s) involved in human CENP-A degradation remain unclear^2^. While Psh1 does not seem to have a mammalian counterpart, Ubr1, Slx5 and Rcy1 are known to have human homologues. It will be of interest to evaluate CENP-A turnover in mammalian cells deficient for these homologues and also to determine whether the human homologues of these E3s are altered in CENP-A-related cancer cells. The knowledge gained will significantly advance our understanding of CENP-A mediated centromere function and related diseases, and may lead to the development of new avenues to control its activity.

## Methods

### Yeast strains and plasmids

Yeast *S. cerevisiae* strains lacking non-essential ubiquitylation components including various E2s and E3s in a BY4741 background were obtained from Dr. Mark Hochstrasser (Yale U.)^29^. Yeast *ubc9* and *ubc1* mutant strains were obtained from Dr. Ray Deshaies (Caltech) and Mark Hochstrasser, respectively. The yeast strain bearing Cse4 tagged with GFP at its endogenous locus was obtained from Dr. Carl Wu (NIH). Strain YHR305 (*PSH1::LEU2 RCY1::KanMX4*) was constructed by replacing *PSH1* with *LEU2* in the *rcy1Δ* strain. Strain YHR317 (*UBR1::URA3 PSH1::LEU2 RCY1::KanMX4*) was constructed by replacing *UBR1* with *URA3* in YHR305. Yeast strain YHR320 (*ubr1Δ PSH1::LEU2 RCY1::KanMX4*) was obtained by popped out *URA3* from YHR317 via FOA selection. Strain YHR331 (*SLX5*::NAT) was constructed by replacing *SLX5* with the *NAT* gene (nourseothricin resistant) in the BY4742 strain. YHR321 (MATa/α *SLX5*::NAT *UBR1*::*URA3 PSH1*::*LEU2 RCY1*::KAN) is a diploid strain constructed by mating YHR317 and YHR331. The haploid strains YHR333 (*SLX5*::NAT *UBR1*::*URA3 PSH1*::*LEU2 RCY1*::KAN), YHR334 (*SLX5*::NAT *PSH1*::*LEU2 RCY1*::KAN), YHR336 (*SLX5*::NAT *UBR1*::*URA3 PSH1*::*LEU2*), and YHR338 (*SLX5*::NAT *PSH1*::*LEU2*) were selected after the sporulation of YHR321. YHR320 (*ubr1Δ PSH1::LEU2 RCY1::KanMX4*), YHR347 (*ubr1Δ SLX5*::NAT *PSH1*::*LEU2 RCY1*::KAN), and YHR349 (*ubr1Δ SLX5*::NAT *PSH1*::*LEU2*) in the BY4741 background were obtained by popping out *URA3* using FOA selection from YHR317, YHR333 and YHR336, respectively.

Yeast cells were grown in rich (YPD) or synthetic media containing standard ingredients and 2% glucose (SD medium), or 2% raffinose (SR medium), or 2% raffinose +2% galactose (SRG medium).

The plasmids pMB1458 bearing 3HA-tagged Cse4 has been previously described^30^. The pRS423-based plasmids expressing wild-type *SLX5* or a RING finger mutant (C561S, C564S) of *SLX5* were kindly provided by Dr. Hochstrasser (Yale U.). The pNT-Flag-UBR1 plasmid bearing Flag-tagged UBR1 was obtained from Dr. Varshavsky (Caltech).

### Ubiquitylation Assay

Yeast cells expressing a *GAL1*-regulated substrate HA-tagged Cse4 or a vector control were grown to log phase in SR medium, and 2% galactose was added to induce protein expression for 4 h. The cells were harvested by centrifugation and lysed with glass beads. The lysates were immunoprecipitated with tandem ubiquitin binding entities (TUBEs) agarose gel (UM401, LifeSensors) for ubiquitylated species at 4 °C for 6 h. The immunoprecipitates were resolved by SDS-PAGE, transferred to PVDF membranes, and immunoblotted with a HA antibody (MMS-101P, BioLegend) to detect ubiquitylated Cse4, which were detected by anti-mouse HRP conjugates and ECL reagents. The samples were also analyzed by anti-HA for Cse4 and anti-Rpt5.

### Expression shut-off assay

As previously described^31^, yeast cells expressing HA-tagged Cse4 were grown at 30 °C to an OD_600_ of ~1 in synthetic medium SR lacking uracil. The expression of HA-Cse4 was induced by galactose for 2 hr^31^. Protein synthesis was turned off by the addition of cycloheximide (150 μg/ml). Samples were collected at the indicated time points. Proteins were extracted by glass bead lysis of cells and processed for immunoblotting with anti-HA (BioLegend), followed by detection with goat anti–mouse HRP conjugate using ECL reagents (GE Healthcare). The stable protein Rpt5 was used as a loading control.

### Antibodies

Antibodies against HA (MMS-101P) and Flag (F1804) were obtained from BioLegend Inc. (San Diego, CA) and Sigma-Aldrich, respectively. The Rpt5 antibody was obtained from Enzo Life Sciences (Farmingdale, NY). The ubiquitin antibody (PA3-16717) was purchased from ThermoFisher Scientific.

## Electronic supplementary material

Supplementary Iformation
