# Supplementary material for: Multiple E3s promote the degradation of histone H3 variant Cse4
Source: Sci Rep. 2017 Aug 17;7:8565. doi: 10.1038/s41598-017-08923-w (PMC5561092; doi:10.1038/s41598-017-08923-w)

Supplementary Information: Source data for immunoblots

Multiple E3s promote the degradation of histone H3 variant Cse4

Haili Cheng[1*], Xin Bao[1], Xin Gan[2], Shiwen Luo[3] and Hai Rao[1*]

From [1]Department of Molecular Medicine, the University of Texas Health Science Center, San Antonio, TX 78229, USA; [2]Research Institute of Respiratory Medicine, the First Affiliated Hospital, Nanchang University, Nanchang, China. [3]Center for Experimental Medicine, the First Affiliated Hospital, Nanchang University, Nanchang, China.

To whom correspondence should be addressed: Haili Cheng or Hai Rao, Department of Molecular Medicine, University of Texas Health, 7703 Floyd Curl Dr., San Antonio, TX 78229, USA. Telephone: 210-562-4149; Fax: 210-562-4161; Email: chengh3@livemail.uthscsa.edu; raoh@uthscsa.edu

Figure 1A

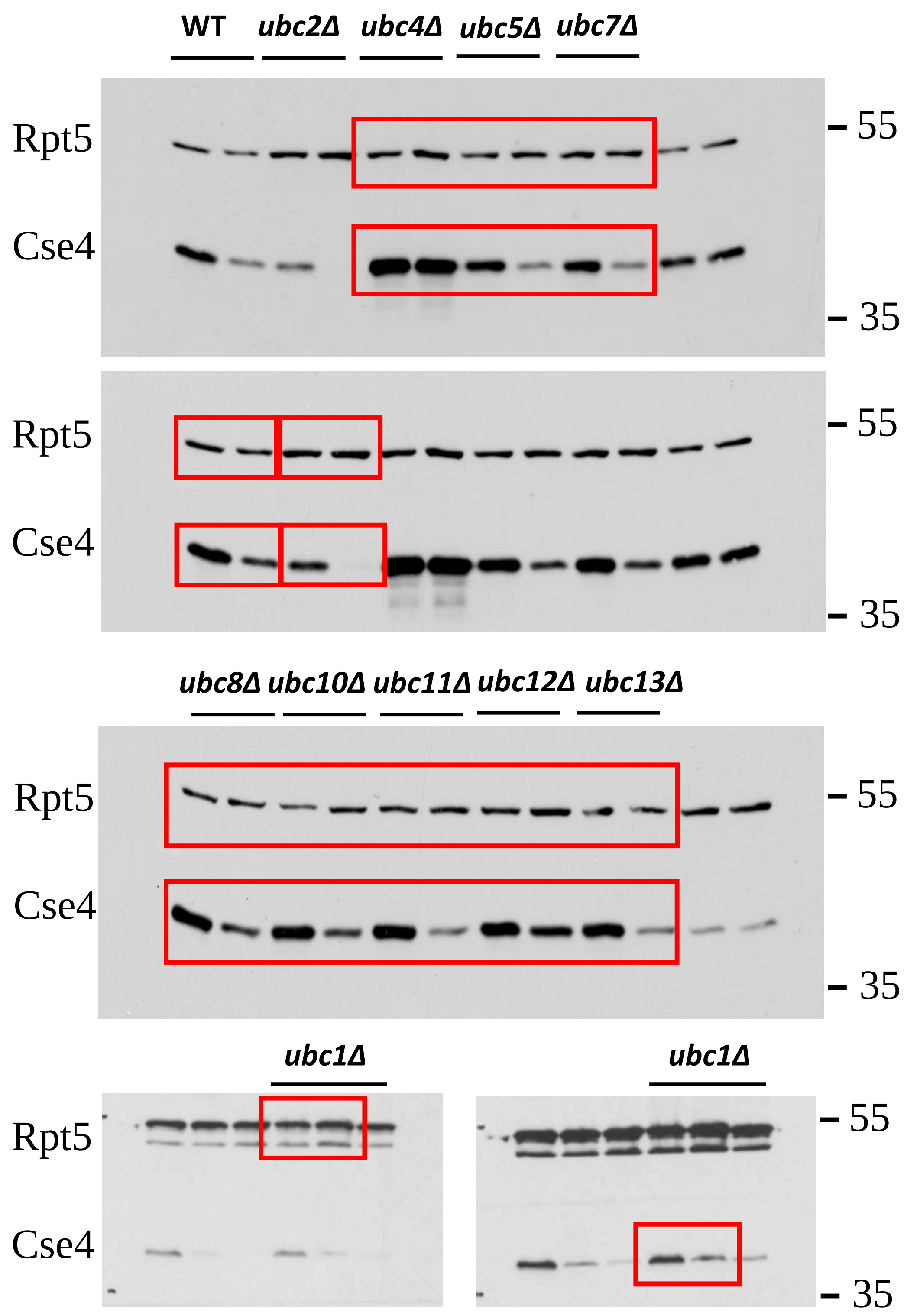

Figure 1B

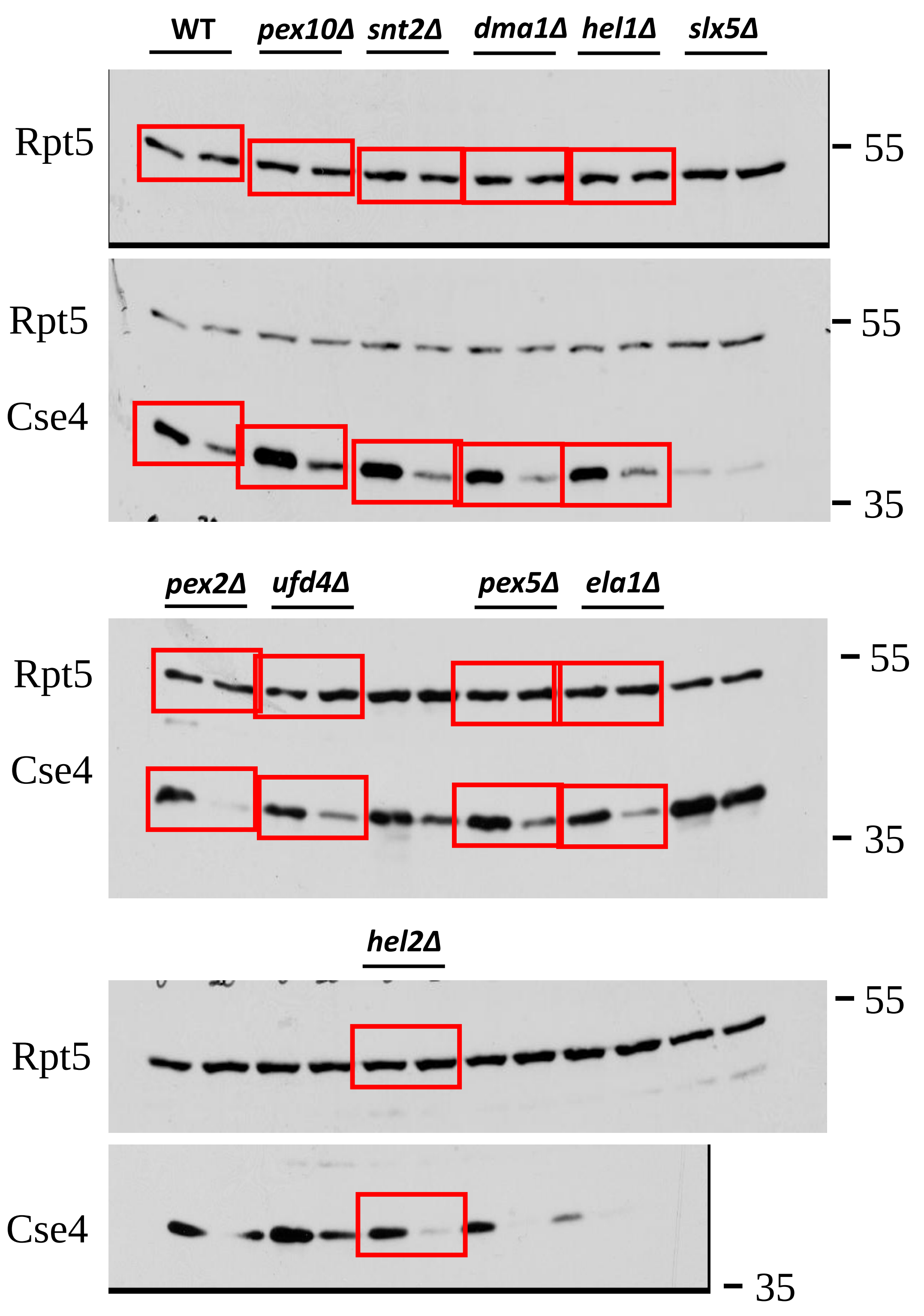

Figure 1B

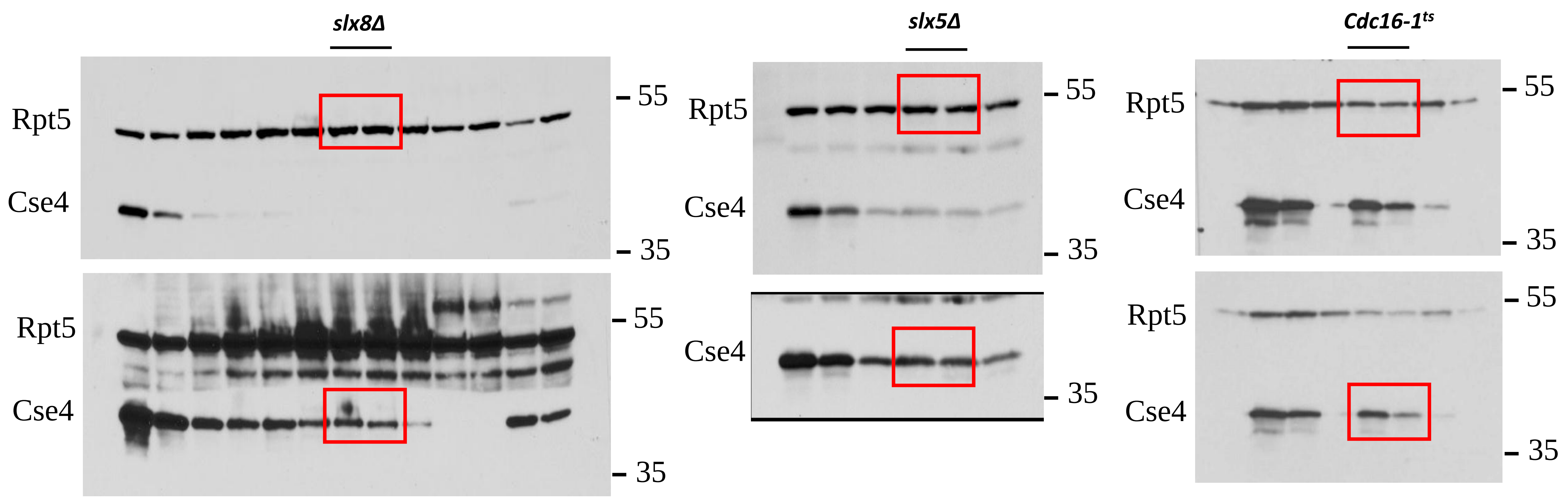

Figure 2A

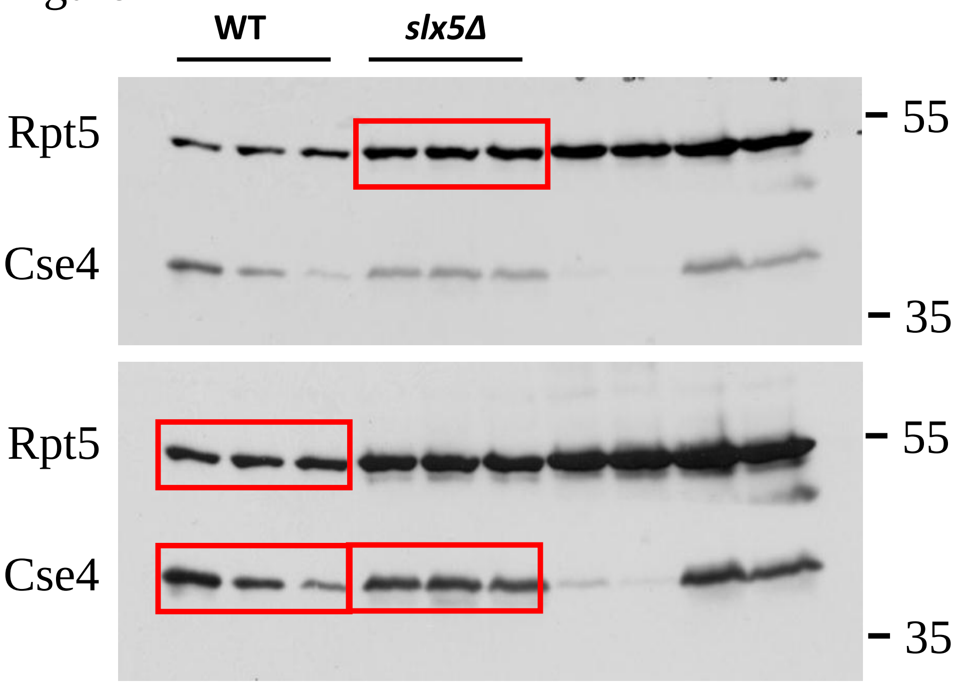

Figure 2C

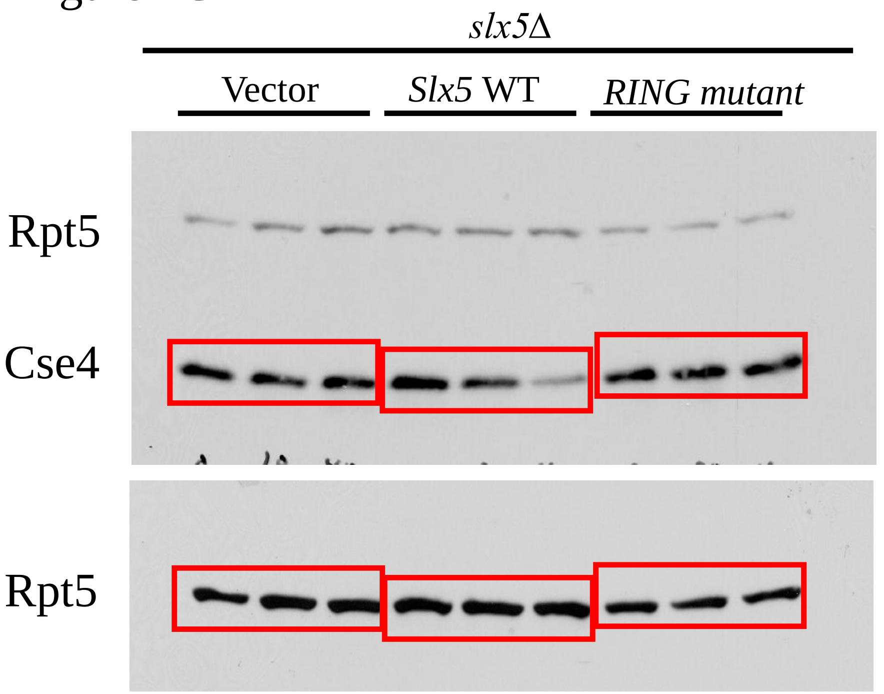

Figure 2E

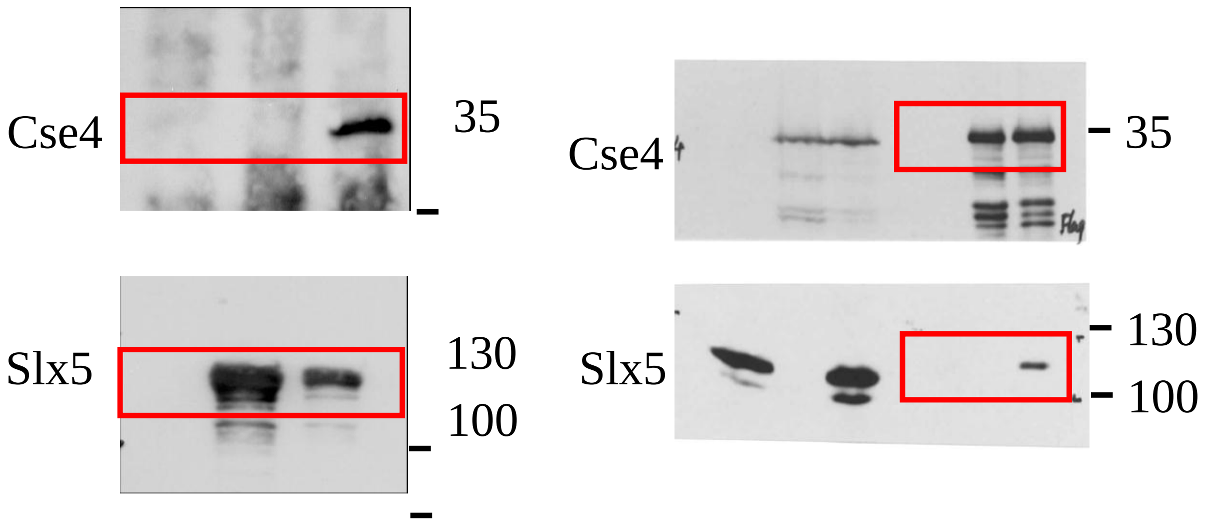

Figure 2F

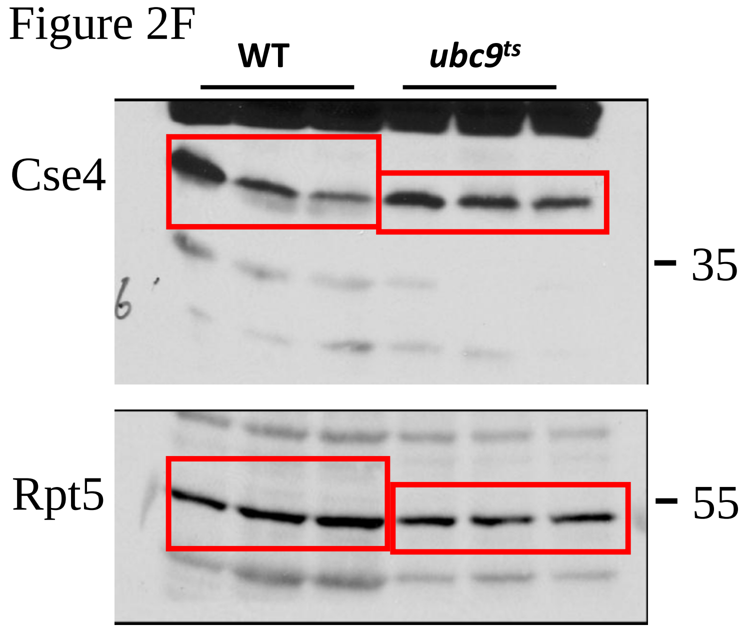

Figure 3A

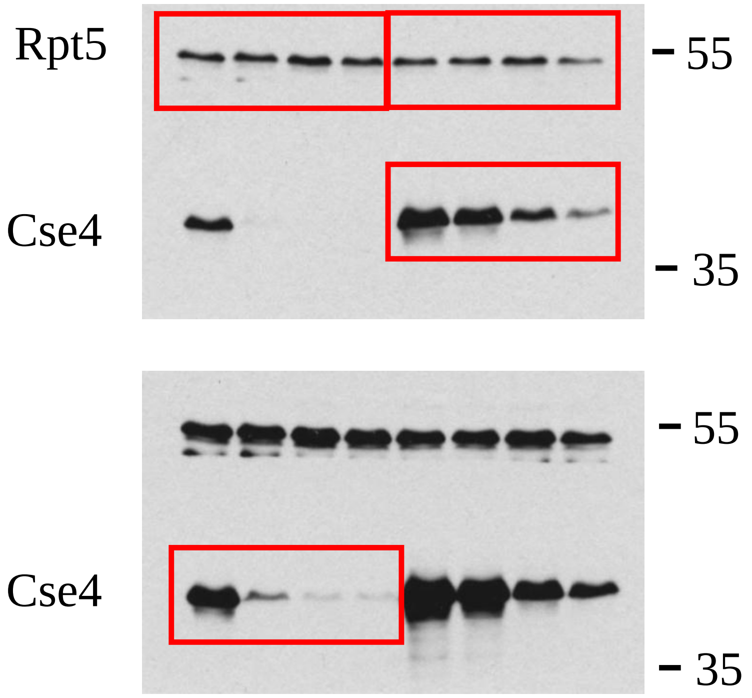

Figure 3C

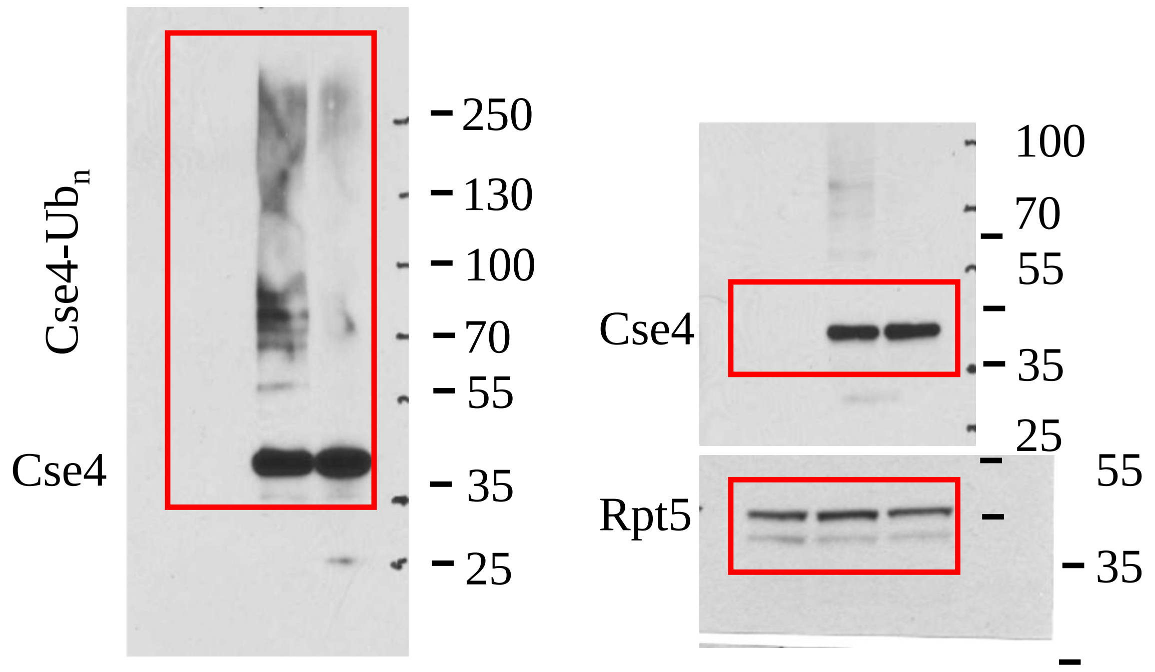

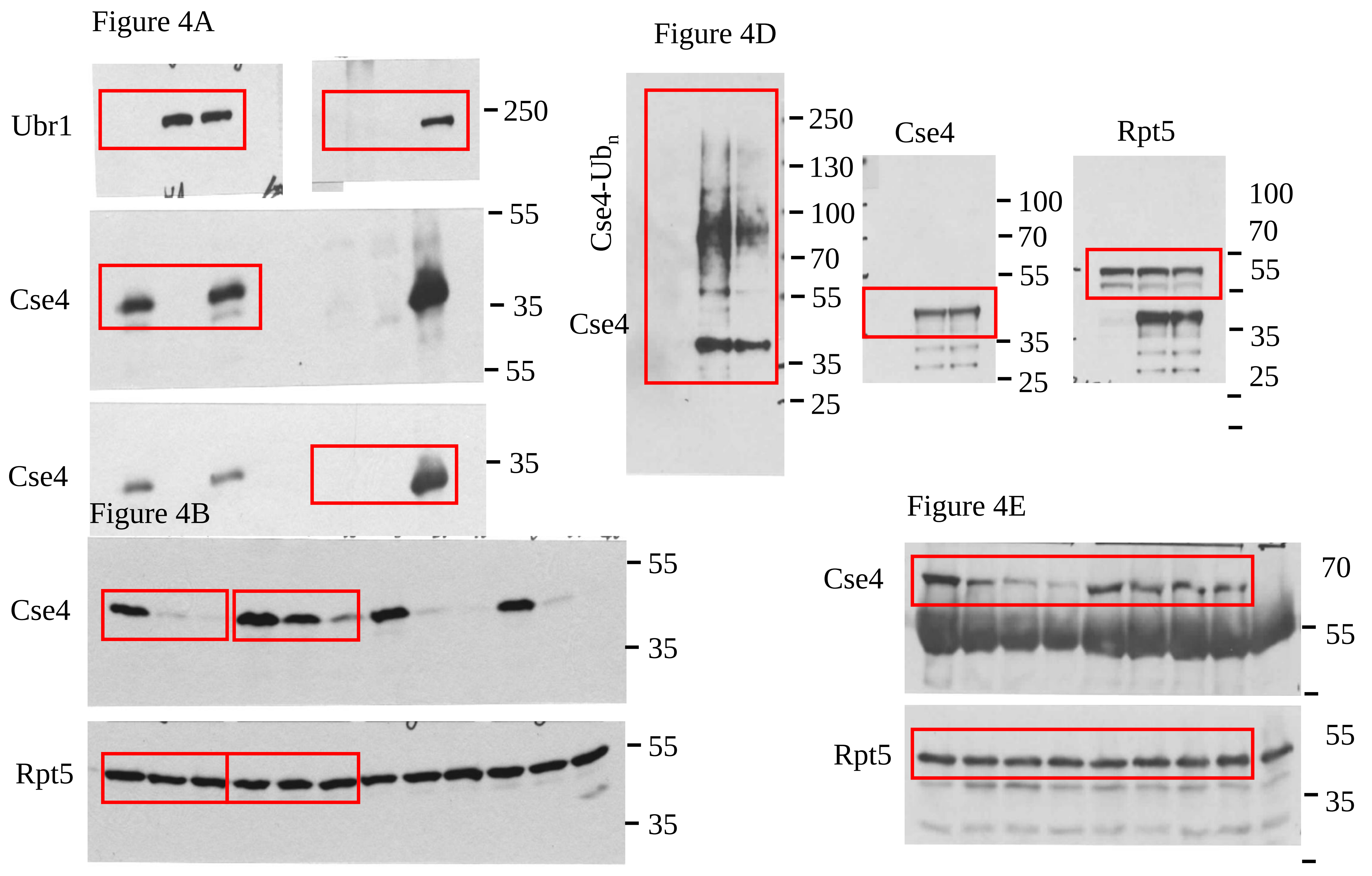
Figure 4A
Ubr1
250
Cse4
55
35
55
Cse4
35
Figure 4B
Cse4
55
35
Rpt5
55
35
Figure 4D
Cse4-Ub$_n$
Cse4
250
130
100
70
55
35
25
Cse4
100
70
55
35
25
Rpt5
100
70
55
35
25
Figure 4E
Cse4
70
55
Rpt5
55
35

Figure 6A

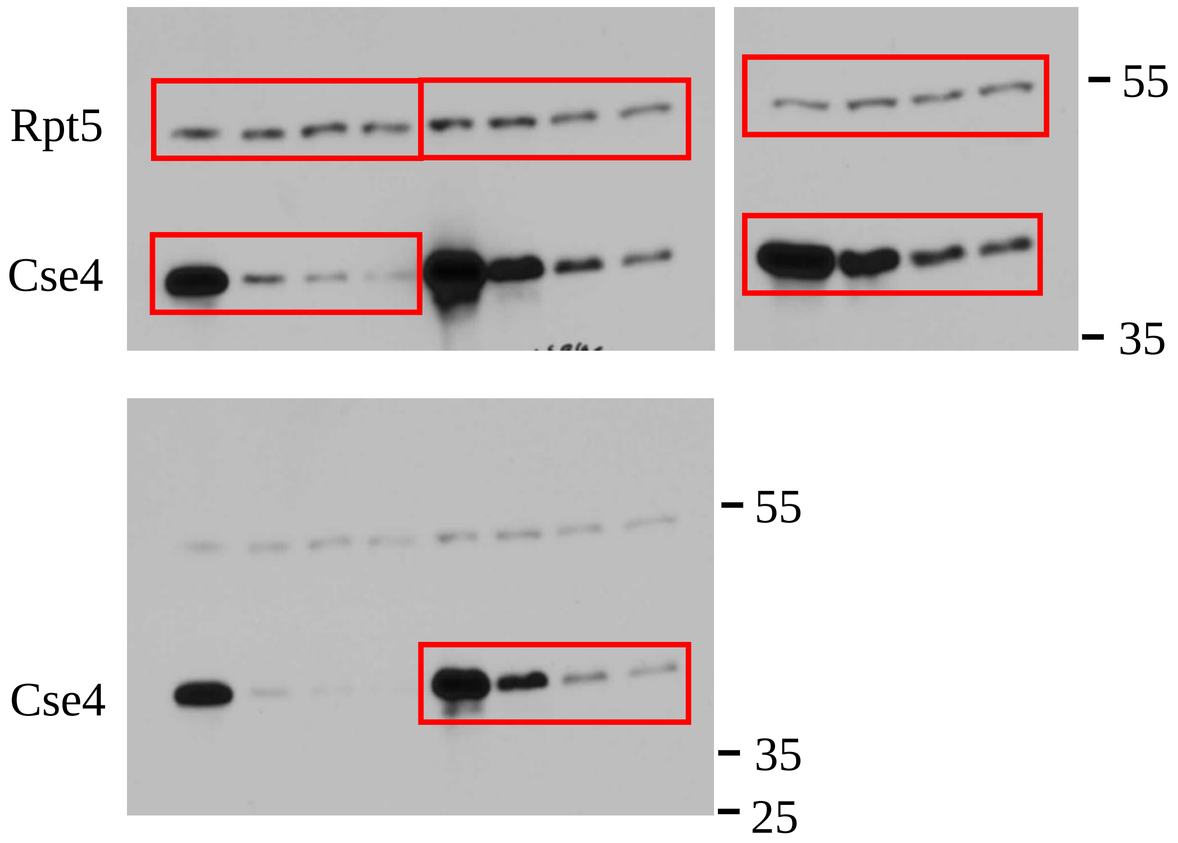

Figure 6B

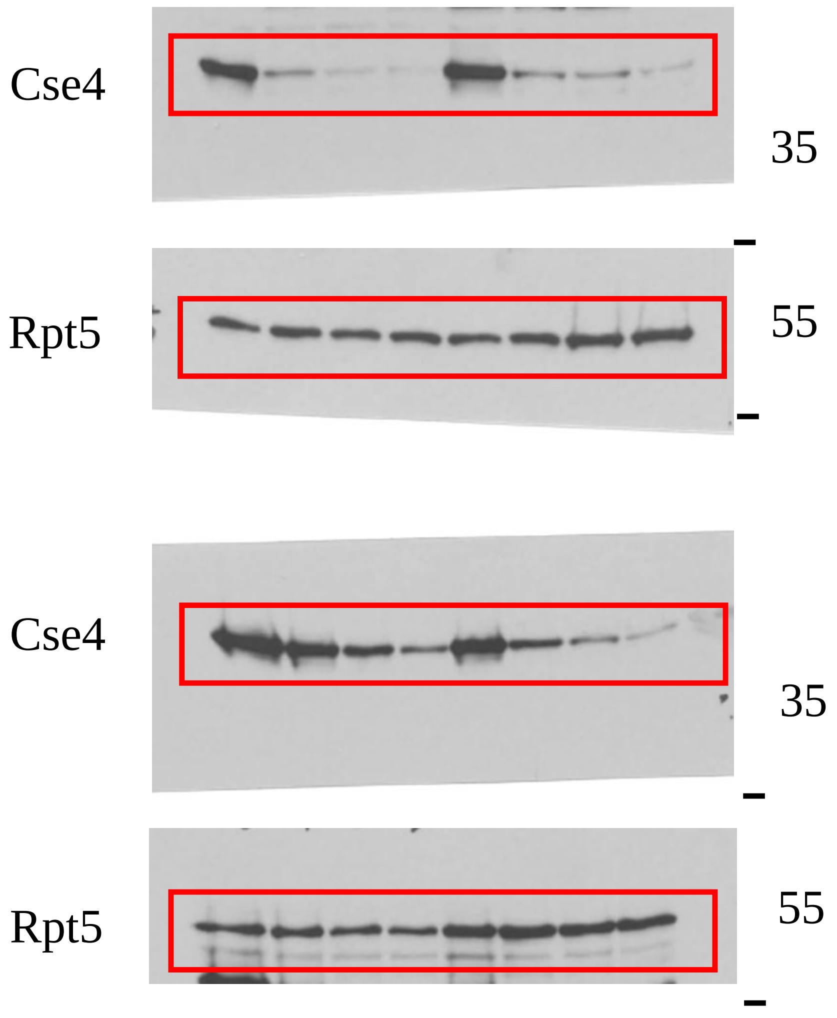

Figure 7A

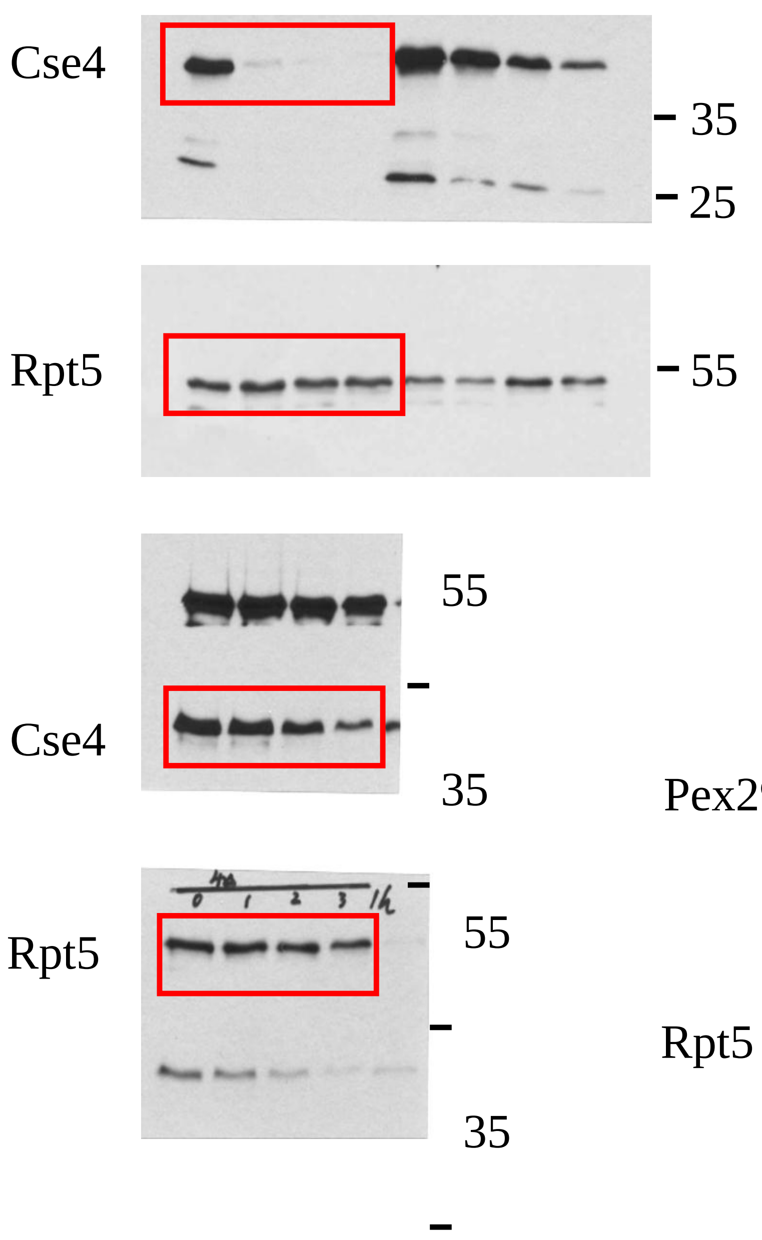

Figure 7C

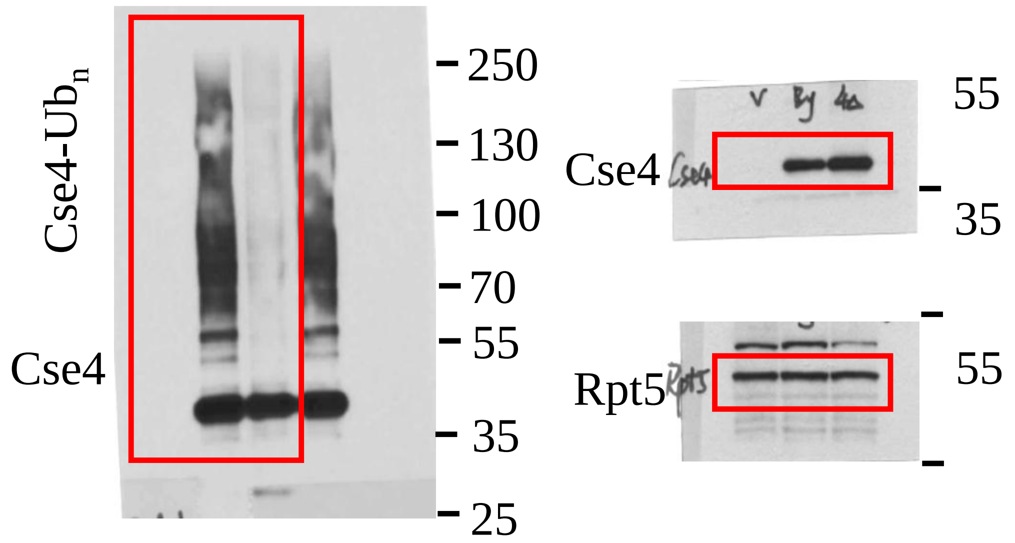

Figure 7D

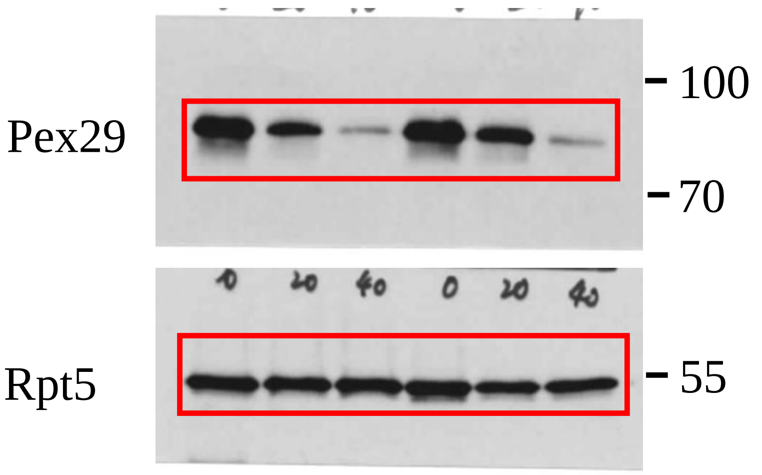

Figure 7E

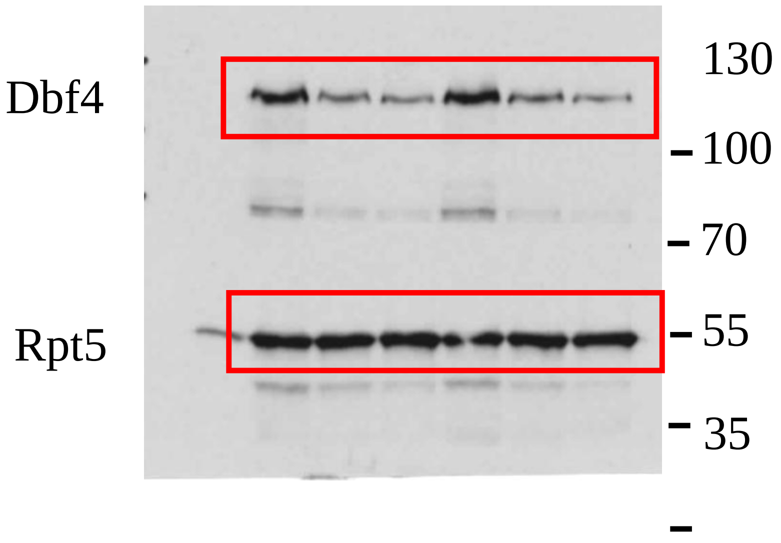

Supplement: Supplementary file 1 — Supplementary Iformation [file 41598_2017_8923_MOESM1_ESM.pdf]
